# Is deep brain imaging on the brink of transformation with a bioluminescence molecule?

**DOI:** 10.1002/bmm2.12115

**Published:** 2024-08-16

**Authors:** Shumao Xu, Farid Manshaii, Jun Chen

**Affiliations:** Department of Bioengineering, University of California, Los Angeles, Los Angeles, USA

**Keywords:** bioluminescence molecule, blood-brain barrier, brain imaging

## Abstract

Cephalofurimazine (CFz), when paired with Antares luciferase, shows superior blood-brain barrier permeability and enhanced imaging depth and clarity for deep brain imaging. This bioluminescence provides a less invasive method for real-time monitoring of deep brain activity, with the potential to advance targeted therapies and deepen our understanding of brain functions. Further molecular engineering and localized delivery can reduce the potential toxicity of CFz and enhance its efficacy for clinical deep brain imaging.

## 1 | INTRODUCTION

Can a single molecule revolutionize how we see the deep brain without invasive procedures? Deep brain spatial information is essential in biomedical imaging for unraveling the intricate mechanisms behind neurological disorders and developing targeted treatments.^[1–5]^ Current deep brain imaging techniques like magnetic resonance imaging (MRI), positron emission tomography (PET), computed tomography (CT), and single-photon emission computed tomography (SPECT) while essential for 3D spatial reconstructions, face challenges in high-efficiency deep brain imaging due to limited spatial resolution, potential artifacts,^[6]^ and the need for high doses of contrast agents or tracers for better signal detection. Bioluminescence imaging has historically provided a glimpse into the live functioning of biological systems, enabling the tracking of gene expression, monitoring disease progression, and studying intricate biochemical pathways in real time.

**FIGURE 1:**
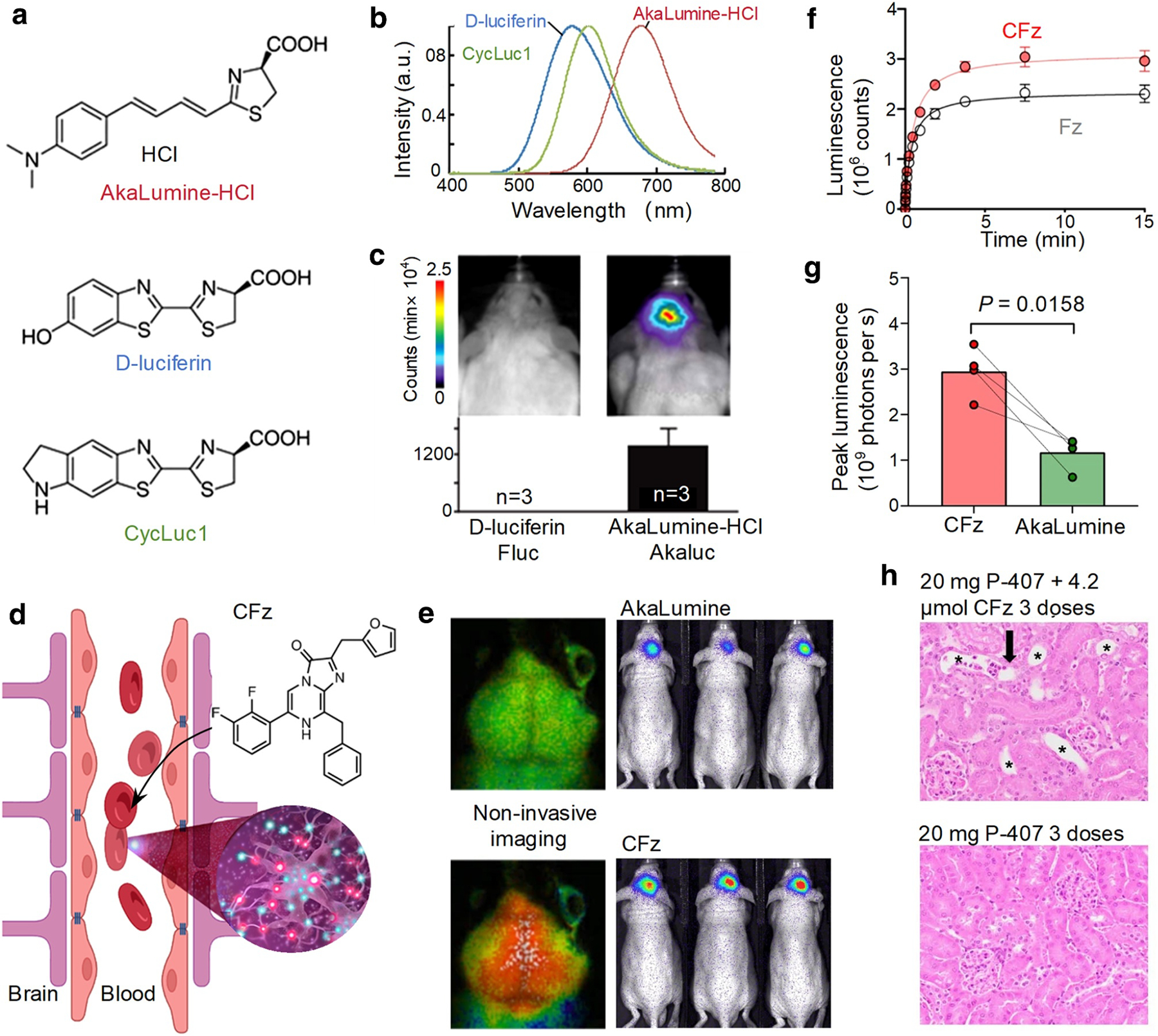
CFz-enhanced deep brain bioluminescent imaging. (a), (b) Common bioluminescence substrates and their emission spectra. Reprinted from ref.^[7]^ Copyright 2016, The Author(s). (c) Bioluminescence images of mice expressing Fluc (left) and Akaluc (right). Reprinted from ref.^[9]^ Copyright 2018, The Author. (d) Illustration of enhanced bioluminescence imaging in deep brain regions with cephalofurimazine (CFz). (e) Non-invasive CFz bioluminescence imaging of brain activity in transgenic mice (left), and comparison of Antares-CFz versus AkaLuc-AkaLumine bioluminescence in co-infected mouse hippocampus (right). (f) Kinetic profiles of Antares luciferase with various substrates. (g) Peak luminescence comparison of Antares-CFz and AkaLuc-AkaLumine. (h) Adverse effect of CFz injection. Kidney: asterisks indicate tubular dilation; arrows show tubular degeneration and luminal sloughing. P-407, Poloxamer-407. e-h, reproduced from ref.^[10]^ Copyright 2023, The Author(s).

Traditional bioluminescent substrates, such as *d*-luciferin (the substrate for firefly luciferase) and CycLuc1, a synthetic analog of *d*-luciferin (Figure 1a), have been widely used for in vivo imaging through their enzymatic oxidation by luciferase to produce light emission, but face limitations in detecting deep tissue targets due to lower sensitivity and suboptimal emission wavelengths (Figure 1b).^[7]^ *d*-luciferin, in particular, has limited solubility and emits light in the visible range, making it more prone to absorption and scattering by biological tissues.^[3]^ To address these challenges, AkaLumine was developed, showing higher sensitivity in detecting deep tissue targets in mouse tumor models compared to *d*-luciferin at the same concentration.^[7]^ To further enhance its applicability for in vivo imaging, AkaLumine-HCl was synthesized, which is soluble in water and emits near-infrared (NIR) bioluminescence (*λ*_max_ = 677 nm) when reacting with Fluc (Figure 1b),^[7,8]^ allowing for deep tissue penetration and improved imaging clarity (Figure 1c).^[9]^ However, AkaLumine still faces limitations in deep brain imaging due to suboptimal blood-brain barrier penetration.

Recent development of cephalofurimazine (CFz) high-efficiency bioluminescent substrates has improved signal brightness and blood-brain barrier penetration, overcoming previous obstacles in monitoring the central nervous system.^[10]^ CFz, a synthetic substrate developed for the NanoLuc luciferase system, has been optimized to capture deep-brain biological processes with enhanced clarity and precision and to cross the blood-brain barrier. The presence of lipophilic aromatic rings and fluoro-substituents in its structure increases lipophilicity, facilitating membrane traversal (Figure 1d). Its compact and planar structure aids in passing through tight junctions while balanced polar functional groups ensure solubility in physiological conditions. Additionally, fluoro-substituents enhance metabolic stability and reduce recognition by efflux transporters. The effectiveness of CFz is evident when paired with Antares luciferase—a bioluminescent reporter that shifts light emission from blue to the orange-red spectrum, enhancing tissue penetration and signal detection in deeper brain regions. This red-shifted bioluminescence is less prone to absorption and scattering, thus providing a clearer image of neuronal activity and biochemical events within the brain. The non-invasive nature of this method is underscored by the retro-orbital administration of CFz in head-fixed mice, allowing for the observation of neural responses similar to those detected by MRI (Figure 1e). CFz has emerged as a frontrunner in brain imaging, outperforming *d*-luciferin, a natural substrate with limited brain accessibility,^[7,9]^ and providing a brighter signal than the synthetic AkaLumine.^[11]^ CFz’s enhanced ability to cross the blood-brain barrier results in a more stable and intense signal. The co-expression of Antares and AkaLuc within the hippocampal region further underscores the substrates’ comparative effectiveness, with CFz outshining Fz in brightness (Figure 1f) and surpassing AkaLumine at various dosages (Figure 1g).

CFz’s enhanced bioluminescent efficiency represents strides in overcoming the longstanding challenges of bioluminescence imaging—specifically, issues related to substrate delivery and signal clarity in the restrictive brain environment. Its ability to cross the blood-brain barrier enables detailed imaging of neuronal activity and other biochemical processes in vivo, providing insights that were previously difficult or impossible to obtain. The breakthrough in CFz-enhanced bioluminescence imaging facilitates real-time, non-invasive imaging of Ca^2+^ levels in genetically defined neurons responding to sensory stimuli. This offers insights into physiological and pathological interactions, disease progression, and treatment efficacy in real time,^[12,13]^ potentially revolutionizing our understanding and treatment of neurological disorders.

## 2 | REDUCING TOXICITY

However, the potential toxicity of CFz at high dosages or with prolonged exposure remains a critical concern (Figure 1h). Daily injections of 1.3 μmol of CFz for up to five days were relatively well-tolerated, but the accumulation of the P-407 excipient in macrophages of the liver and spleen indicated potential long-term effects. The effectiveness and safety of CFz-based bioluminescence imaging may also vary depending on the route of administration. Intraperitoneal (i.p.) injections were found to produce brighter signals compared to subcutaneous injections, but they also posed risks of toxicity at higher doses. Its synthetic structure, designed for high permeability and brightness, necessitates long-term toxicity studies to establish safe dosage thresholds for repeated use in humans.^[14]^

To mitigate this concern, several potential strategies can be employed to reduce toxicity and enhance safety. Optimizing the chemical structure of CFz by adjusting the balance between lipophilic and hydrophilic properties can potentially improve its ability to cross the blood-brain barrier while minimizing interactions with non-target tissues. Incorporating biocompatible and metabolically stable groups, such as polyethylene glycol (PEG) chains, can enhance solubility and reduce immunogenicity. Additionally, developing CFz as an “enzyme-triggered prodrug” can ensure activation only at the target site, minimizing systemic exposure and enhancing safety.^[15]^ Meanwhile, “targeted biodegradable nanoparticle delivery”, using poly(lactic-co-glycolic acid) (PLGA) or liposomes functionalized with ligands like transferrin or lactoferrin, can also be developed to protect CFz from premature degradation and reduce direct exposure to non-target tissues, and ensure precise brain targeting.^[16,17]^ Further controlled release from these nanoparticles can maintain a localized and sustained delivery of CFz, reducing peak plasma levels that could lead to toxicity.^[18]^ Moreover, genetic engineering approaches, including optogenetic techniques, CRISPR/Cas9, and inducible systems, can ensure localized, controlled, and temporally precise expression of luciferase,^[19–22]^ thereby reducing the need for high systemic levels of CFz and minimizing prolonged exposure and cumulative toxicity.

## 3 | ENHANCING SPECIFICITY

Although CFz can cross the blood-brain barrier more effectively than previous substrates, achieving consistent delivery across various brain regions and pathological states remains a challenge. Variability in bioluminescence signals due to hemodynamic responses, particularly during high-frequency imaging intervals (<10 s), can complicate the interpretation of time-lapse imaging data, as CFz-based imaging relies on blood circulation to deliver the substrate and oxygen, leading to potential confounding effects from hemodynamic fluctuations rather than true changes in luciferase activity, especially during brain imaging where neuronal activity rapidly alters blood flow. Additionally, the fast kinetics of CFz may limit its use in timelapse imaging, where the slower-decaying signals of AkaLuc–AkaLumine are more advantageous.

Enhanced specificity can minimize off-target effects and background noise,^[6,23]^ interfering with the accurate interpretation of bioluminescence signals. This can be potentially achieved by optimizing the CFz substrate’s structure to fit into the NanoLuc luciferase’s binding pocket by adjusting the substrate’s size, shape, and electronic properties to increase affinity and reduce non-specific interactions. Additionally, incorporating modifications that align with the NanoLuc binding pocket, such as hydrophobic groups for enhancing binding affinity and steric hindrance to prevent interactions with endogenous enzymes, proteins, and small molecules, reduces non-specific activation or background signals.^[15]^ By reducing these interactions, the bioluminescence signal is ensured to result solely from the reaction between CFz and NanoLuc, thereby improving the imaging accuracy. Meanwhile, encapsulating CFz in nanoparticles or liposomes functionalized with ligands^[24]^ or antibodies targeting NanoLuc-expressing regions, as well as attaching CFz to neuron-specific antibodies^[25]^ that naturally cross the blood-brain barrier, can achieve precise delivery and further enhance specificity.

The emergence of CFz in synergy with Antares luciferase has been transformative in bioluminescence imaging, offering unparalleled clarity in visualizing brain function and promising insights into the brain’s complexity. Its advantages over traditional substrates like *d*-luciferin and AkaLumine establish CFz as a superior tool for non-invasive deep-brain imaging, potentially reshaping our approach to studying and monitoring the brain’s intricate functions. By molecular engineering and localized delivery, the potential toxicity of CFz can be reduced, enhancing its safety and efficacy for clinical applications in deep brain imaging.
